# A glance at the applications of Singular Spectrum Analysis in gene expression data

**DOI:** 10.1016/j.bdq.2015.04.001

**Published:** 2015-05-29

**Authors:** Hossein Hassani, Zara Ghodsi

**Affiliations:** a Institute for International Energy Studies (I.I.E.S), 1967743711 Tehran, Iran; b The Statistical Research Centre, Bournemouth University, UK

**Keywords:** Singular Spectrum Analysis, Filtering, Signal extraction

## Abstract

In recent years Singular Spectrum Analysis (SSA) has been used to solve many biomedical issues and is currently accepted as a potential technique in quantitative genetics studies. Presented in this article is a review of recent published genetics studies which have taken advantage of SSA. Since Singular Value Decomposition (SVD) is an important stage of this technique which can also be used as an independent analytical method in gene expression data, we also briefly touch upon some areas of the application of SVD. The review finds that at present, the most prominent area of applying SSA in genetics is filtering and signal extraction, which proves that SSA can be considered as a valuable aid and promising method for genetics analysis.

## 1 Introduction

Nowadays there exists a large amount of datasets in the field of genetics and expression measurement and there are many different methods and techniques for analysing these datasets [1], [2], [3]. However, it has been widely accepted that the major difficulties in working with such data no longer is the validity of expression measurements, but the reliability of inferences from the data as the achieved data is difficult to understand without the use of proper analytical tools [4] and if research result is obtained from an inappropriate model it can never be translated into a valid scientific context [5].

Historically, such data have been analysed using parametric methods [6], [7]. However, constraining pre-assumptions needed for parametric approaches led towards the growing popularity of nonparametric methods. Recently it has been concluded that non-parametric techniques can be used as an alternative approach for analysing genetics data because of their inherent nature [8], and accordingly the applications in biomedical and genetics fields have expanded.

Among many non-parametric methods, Singular Spectrum Analysis (SSA) is a relatively new approach which has proven to be very successful. SSA has already transformed itself into a standard tool in the analysis of biomedical, mathematical, geometrical and several other time series [9], [10], [11] and recently it has also been applied in genetics which has illustrated its strong potential for such studies [12], [13].

The emergence of SSA is usually associated with the work by Broomhead in 1986 [14]. However, the ideas of SSA were independently developed in Russia and in several groups in the UK and USA. Since after, several papers on the methodology and applications of SSA have been published (see, for example, [15], [16]). An introduction to this technique can be identified in the paper by Elsner and Tsonis [17] and a comprehensive description with several examples of theoretical and practical aspects of SSA can also be found [8], [18].

The main advantages of the SSA technique in the field of genetics can be attributed to its signal extraction and filtering capabilities [19], batch processing of a set of similar series [20] and derivation of an analytical formula of the signal [21]. The application of SSA for noise reduction in microarrays, and signal extraction in gene expression data has received more interest. The reason underlying the significant interest in the SSA technique's filtering capabilities are due to the fact that genetics data is often characterised by the existence of considerable noise, filtering this noisy data is considered as one of the most arduous tasks when analysing genetics data [22], [23].

For example, microarray is a very useful method for acquiring quantitative data in genetics and researchers today are conducting most of their studies using this method. The main advantage of microarray is the capability of studying thousands of genes simultaneously. However, microarray data usually contains a high level of noise, which can reduce the performance of the results [24].

This article categorises and summarises almost all recently published articles associated with the application of SSA in genetics.

Presented below, is a short description of SSA technique in doing so we mainly follow [9] where a more detailed description is made available. Moreover, the R package for this technique including decomposition, forecasting and gap filling for univariate and multivariate time series can be downloaded via [25]

Consider a set of genetics observations in a series of *Y*_*N*_ = (*y*_1_, …, *y*_*N*_) with length of *N*. After choosing a window length *L* where (2 ≤ *L* ≤ *N* − 1), we construct the *L*-lagged vectors *X*_*j*_ = (*y*_*j*_, …, *y*_*L*+*j*−1_)^*T*^, *j* = 1, …, *K* where *K* = *N* − *L* + 1. Define the matrix $\text{X}={\left(x_{\mathrm{ij}}\right)}_{i,j=1}^{L,K}=(X1,\ldots ,X_{K})$. Now **X** is our multivariate data with *L* characteristics and *K* observations. The columns *X*_*j*_ of **X**, are the vectors, positioned in an *L*-dimensional space ${\mathbb{R}}^{L}$. Define the matrix **XX**^*T*^: SVD of **XX**^*T*^ gives us the collections of *L* eigenvalues (*λ*_1_ ≥ ⋯ ≥ *λ*_*L*_ ≥ 0) and the corresponding eigenvectors *U*_1_ … *U*_*L*_ where *U*_*i*_ is the normalised eigenvector corresponding to the eigenvalue *λ*_*i*_(*i* = 1, …, *L*). A group of *r* (with 1 ≤ *r* < *L*) eigenvectors determines an *r*-dimensional hyperplane in the L-dimensional space RL of vectors *X*_*j*_. By choosing the first *r* eigenvectors *U*_1_, …, *U*_*r*_, then the squared *L*_2_-distance between this projection and **X** is equal to $\Sigma_{j=r+1}^{L}\lambda_{j}$. Based on the SSA process, the *L*-dimensional data are projected onto this *r*-dimensional subspace and the final diagonal averaging gives us an appropriate approximation of the first one dimensional series.

The remainder of this paper is organised as follows. In the following section we present a review of papers involving the application of SSA and SVD^1^ on signal extraction and noise reduction. The SSA based on minimum variance and a hybrid modelling approach combining SSA and autoregressive (AR) model are also discussed in depth in that section. Section 3 provides theoretical developments leading to what is termed as “two-dimensional SSA” and the paper ends with some conclusions in Section 4.

## 2 Signal extraction and filtering

In this section we identify existing applications of SSA for signal extraction and noise filtering in genetics.

The first such application is reported in 2006 where SSA was used for signal extraction of *Drosophila melanogaster*'s gene expression profile [19]. The idea of using SSA for signal extraction was then followed in an approximately similar study in [26] which led to an improved result. By 2008 a more technical study conducted on the methodology of signal extraction from the noisy *Bicoid (Bcd)* protein profile in *Drosophila melanogaster* was presented in [21]. The problem under investigation in that study was complicated by two facts: (i) the data contained outliers and (ii) that the data was exceedingly noisy and the noise consisted of an unknown structure. The author examined two approaches for reconstructing signal more precisely: the use of small window length and improvements to separability by adding a constant to the series.

In addition, the activation of the *hunchback (hb)* gene in response to different concentrations of *Bcd* gradient was studied in [27] and SSA was applied for filtering two kinds of noise; experimental noise and the noise caused by variability in nuclear order [27].

### 2.1 SSA based on minimum variance

Recently, a modified version of SSA was examined for filtering and extracting the *bcd* gene expression signal [28] and the results illustrated that SSA based on minimum variance can significantly outperform the previous methods used for filtering noisy *Bcd* [28].

SSA based on minimum variance mainly relies on the concept that by dividing the given noisy time series into the mutually orthogonal noise and signal+noise components, an enhanced estimation of the signal can be achieved. Thus, after performing SVD, by adapting the weights for different obtained singular components, an estimation of the Hankel matrix **X**, will be achieved which in turn is corresponds to a filtered series.

A short description of the SSA based on minimum variance is given below. For more details, see [28], [29].

Let us begin with the Singular Value Decomposition (SVD) of the trajectory matrix **X**:

(1) $$\text{X}=\text{U}(\text{W}\Sigma ){\text{V}}^{T},$$

where **W** is the diagonal matrix of the weights to be determined. The SVD of the matrix **X** can be written as:

(2) $$\text{X}=[{\text{U}}_{1}\;\;\;\;{\text{U}}_{2}]\left[\begin{array}{cc} \Sigma_{1} & 0\\ 0 & \Sigma_{2} \end{array}\right]\left[\begin{array}{c} {\text{V}}_{1}^{T}\\ {\text{V}}_{2}^{T} \end{array}\right],$$

where ${\text{U}}_{1}\in {\mathbb{R}}^{L\times r}$, $\Sigma_{1}\in {\mathbb{R}}^{r\times r}$ and ${\text{V}}_{1}\in {\mathbb{R}}^{K\times r}$.

Now, the issue is in selecting the weight matrix **W**. If we represent the SVD of the Hankel matrix related to the signal as **S**, by considering different criteria in choosing this matrix, different estimation of **S** can be achieved. The LS Estimate of **S** is the current widely used approach in selecting the weight matrix **W**. This approach is based on the idea of removing the noise subspace but keeping the noisy signal uncorrelated in the signal+noise subspace. However, the accuracy of this estimator is mainly dependent on the estimation of the signal rank *r* since selecting singular values in LS follows a binary approach. Although in using this estimator, considering any assumptions is not needed.

In MV Estimate of **S** proposed by Hassani in [29], removing the noise components in the signal + noise subspace has been improved considerably. However, to obtain the MV estimate considering some assumptions is essential (for more details see [29]).

Let us now consider the weight matrix **W** of the LS and MV estimates:

(3) $$\begin{array}{c} {\hat{\text{S}}}_{\mathrm{LS}}={\text{U}}_{1}({\text{W}}_{\mathrm{LS}}\Sigma_{1}){\text{V}}_{1}^{T}\\ {\hat{\text{S}}}_{\mathrm{MV}}={\text{U}}_{1}({\text{W}}_{\mathrm{MV}}\Sigma_{1}){\text{V}}_{1}^{T}, \end{array}$$

where

(4) $$\begin{array}{c} {\text{W}}_{\mathrm{LS}}={\text{I}}_{r\times r}\\ {\text{W}}_{\mathrm{MV}}=\mathrm{diag}\left(\left(1-\frac{\sigma_{\mathrm{noise}}^{2}}{\lambda_{1}^{2}}\right),\ldots ,\left(1-\frac{\sigma_{\mathrm{noise}}^{2}}{\lambda_{r}^{2}}\right)\right). \end{array}$$

It should be noted that, **U**_1_ and **V**_1_, of LS and MV estimates are the same, but the singular values are different.

**Fig. 1:**
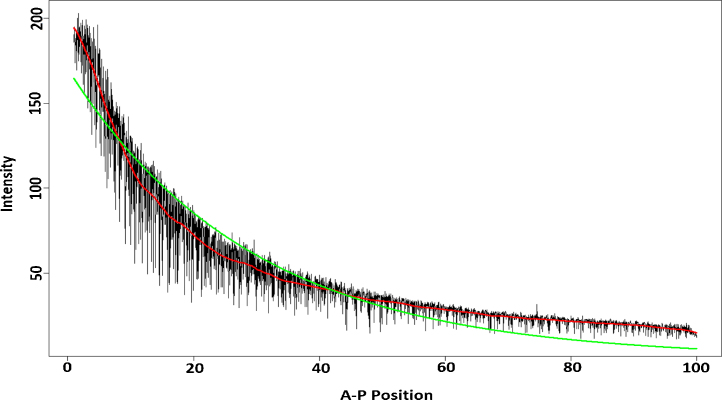
Temporal dynamics of *Bcd* expression, cleavage cycle 14(6). Red and green lines show the trend extracted by *SSA*_*MV*_ and *SDD*[28]. (For interpretation of the references to colour in this figure legend, the reader is referred to the web version of the article.)

Fig. 1 shows the signal extracted by SSA-MV in [28] along with fitted trend obtained by Synthesis Diffusion Degradation (SDD) model as the most widely accepted model in analysing *Bcd* gradient. As it appears SSA-MV yields more promising result for *Bcd* signal extraction.

### 2.2 SSA combined with AR model

Among many applications of the microarray technique, the study of rhythmic cellular processes has been considered as an important application. Rhythmic cellular processes are mainly regulated by different gene products, and can be measured by using multiple DNA microarray experiments. Having a group of gene experiments over a time period, a time series of gene expression related to the rhythmic behaviour of that specific gene will be achieved. Several studies on extracting the regulatory information from time-series microarray data and detection of cyclic and non-uniformly sampled short time series of gene expressions can be found in [30], [31], [32]. The characteristics of the rhythmic gene expression is as follows [33]: the number of time points and cycles related to a profile is usually very few. For example, the 14 time point elutriation observations may be in constitution of just one cell-cycle [20], this data set usually contains many missing values which need to be determined [34], the intervals spaced between time points are not equal and the gene expression data is extremely noisy [33].

**Fig. 2:**
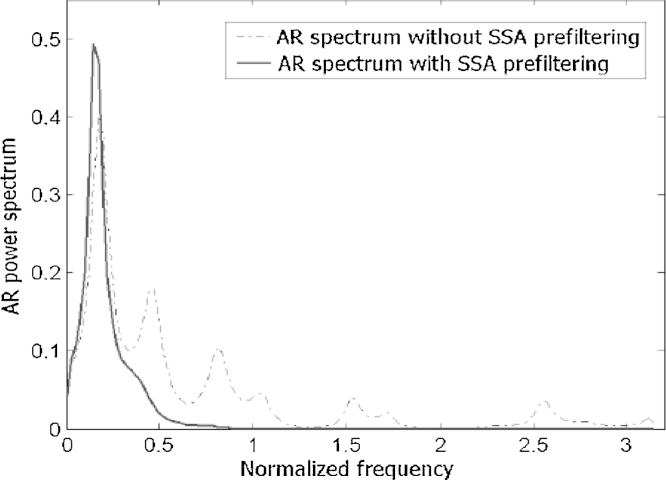
The AR spectra of the expression profile of Dihydrofolate Reductase–Thymidylate Synthase with and without SSA filtering [12].

In 2008, Du et al. implied SSA for analysing microarray results for extracting the dominant trend from noisy expression profiles and reducing the effect of noise [12]. The authors have also proposed a new procedure for analysing the periodicity of the transcriptome of the intraerythrocytic developmental cycle (IDC) by combining autoregressive (AR) model and SSA. This combination enabled them to successfully identify almost 90% of genes (4496 periodic profiles) in *P. falciparum*, which was a considerable achievement in terms of detecting 777 additional periodic genes in comparison to the results in [35]. Fig. 2 depicts the improvement yielded by using SSA in analysing periodicity. As shown in this figure, due to the removal of noise using the SSA, spurious peaks are eliminated from the spectrum.

Four subsequent research works followed this procedure and successfully improved the capability of detecting periodicity from 60% to 80% [36], [37], [38], [39].

According to [33], the periodic profiles can be detected by combining SSA and AR as follows:

• At the first step SSA is used for the reconstruction of each expression data. For this aim only those expression profiles with sum of first two eigenvalues over the sum of all eigenvalues greater than 0.6 are selected for reconstruction.
• The second step is devoted to the calculation of the AR spectrum, frequency *f*_*i*_ at peak value point and the ratio of the power in *f*_*i*_ Regions of Interest (ROI) of the reconstructed profiles achieved in the first step. It should be noted that to obtain the ratio between the power of the signal within the frequency band [*f*_*i*−1_, *f*_*i*+1_] and the total power we follow *S* = *power*_*i*_/*power*_*total*_.
• If the obtained power ratio *S* is larger than 0.7, the corresponding profile would be classified as periodic [33].

Presented below is a summary of the theory combining SSA and AR based on [39]:

Let the microarray data be *g* × *N* matrix (*g* ≫ *N*), where *g* is the number of gene expression profiles and *N* corresponds to the number of samples. Time series gene expression *Y*_*N*_ = (*y*_1_, …, *y*_*N*_) can then be written in a form of an *AR*_(*p*)_ model by forward-backward linear prediction as follows [39]:

(5) $$y_{i}=\alpha_{1}y_{i-1}+\alpha_{2}y_{i-2}+\cdots +\alpha_{p}y_{i-p}\;(i=p+1,\ldots ,N).$$

Using the forward prediction linear system the AR coefficients (*α*_1_, *α*_2_, …, *α*_*p*_) can be estimated and gene expression can be recognised as a linear system. In an *AR*_(*p*)_ model, *p* has to be set in a way that the system models the desired trend and avoids redundant data such as noise. By choosing *m* gene expression profiles, the number of linear equations is equal to *m* × 2(*N* − *p*).

As mentioned above, the SSA technique is often applied prior to spectral analysis, as a filtering method, in order to achieve a better accuracy. This is done by ignoring the small singular values in the reconstruction stage. Removing the noise component from the original noisy signal *Y*_*N*_ yielding the noise reduced series ${\hat{Y}}_{N}$ which can consequently be used for estimating the AR coefficients *α*_*i*_ (*i* = 1, …, *p*), and ultimately estimated *α*_*i*_ are obtained using Yule–Walker equations [39].

### 2.3 SVD

Although SVD is an stage of SSA technique, it has also been used independently as a very useful and applicable tool for analysing the microarrays data (see for example, [40]) in which the challenging problem of eliminating cross hybridization in real-time microarray data was considered. Vikalo et al. in [40] evaluated multiple techniques such as SVD for separating the components of the compound signal, to find a better estimation of the amounts of both hybridising and cross-hybridising targets.

However, the microarray technology has become increasingly affordable, the data is still very complicated to analyse. In 2010 Rau et al. in [41] presented an algorithm to infer the structure of gene regulatory networks using an empirical Bayes estimation procedure for the hyperparameters of a linear feedback state-space model and used SVD of the block-Hankel matrix for model selection. This approach enabled them to significantly reduce the computation time required for running the algorithm, as it eliminates the need to run the algorithm over a wide range of values for the hidden state dimension. Moreover, as discussed in [42] the numerical computations in SVD even for a huge data set such as microarray data is not time extensive. In that study a new approach for ranking genes is presented which is based on the genes degree of regulation and the authors showed that the ranking of genes according to regulation is genetically more meaningful rather than using the absolute expression or variation over time and this method can be a valuable aid in finding the regulatory pathways and networks. In that work SVD was performed to the block-Hankel matrix of observation autocovariances estimated from the gene expression data, and the number of singular values related to the large magnitude was performed as an estimate for the best state space dimension. The singular values of the estimated Hankel autocovariance matrix were calculated and standardised to a 0–1 scale. The number of singular values of magnitude larger than the threshold was used as an estimate for the state space dimension.

Based on the mentioned studies, SSA procedure is a flexible technique for the signal extraction and gene expression degradation modelling. The feasibility of capturing the signal from segmentation genes profile in *Drosophila* embryos and studying the rhythmic behaviour of a specific gene suggest that this technique may be of general use in evaluating other expressional systems. Thus, this method can be a valuable aid in analysing spatially inhomogeneous noisy data.

## 3 Application of two dimensional SSA

The 2D-SSA approach has been used to process two-dimensional scalar fields [8]. The first difference between 2D-SSA and SSA is about the window length. In 2D-SSA analysis we need to choose two different values for the window length L (*L*_1_, *L*_2_), whilst in univariate SSA we only require to select one window length. Note that if *L*_2_ = 1, then 2D-SSA is equivalent to SSA. By choosing *L*_2_ = *M*, the interaction among different series is taken into account [43]. For more information see [44].

It is worthy to mention that the gene expression can be traced either in just anterior–posterior (AP) axis or both AP and dorso-ventral (DV) axis which the latter case is the subject to 2D-SSA. The data points used in 2D-SSA study attributes to the intensity levels for the positions along both AP and DV axis and are considered as a sequenced series.

*Bcd* is a transcriptional regulator of downstream segmentation genes where the alteration in *Bcd* gradient shifts the downstream patterns [45]. However, it has been accepted that in the embryos, zygotic gene products are considerably more precisely positioned than the gradients related to maternal genes, which indicates an embryonic error depletion process [46]. In 2011 the anterior–posterior (AP) segmentation of *Drosophila* was studied in [47] to determine how gene regulation dynamics control noise. In this research the activation of the *hb* gene by the *Bcd* protein gradient in the anterior part was studied by modelling the noise observed in *hb* regulation using a chemical master equation approach. For solving this model, the MesoRD software package has been used which mainly follows a stochastic approach [48] and the results indicate that *Hb* output noise is mostly dependent on the transcription and translation dynamics of its own expression, and that the multiple *Bcd* binding sites located in the *hb* promoter also improve pattern formation[49].

Noise in that study is calculated by:

(6) $$\sqrt{\frac{\sum {\left[(\mathrm{data}-\mathrm{trend})/\mathrm{trend}\right]}^{2}}{m-1}},$$

where data is background-removed intensity of an energid (nucleus plus surrounding cytoplasm), trend is the signal extracted using 2D SSA (AP and DV) and *m* indicates the positions. This measurement is obtained for the activated region (15–45% EL).

In 2012 Golyandina et al. used 2D SSA to measure between-nucleus variability (noise) seen in the gradient of *Bcd* in *Drosophila* embryos [50]. In that paper, they measured the noise using 2D SSA for comparing the results of fixed immunostained embryos with live embryos with fluorescent *bcd* (bcd-GFP).

It should be noted that using SSA for signal extraction gives the ability of using both dimensions (AP and DV) which leads to a more reliable result as in this case more detailed information regarding the data has been considered by the technique to give the results.

## 4 Conclusion

The aim of this paper was devoted to review the applications of SSA in genetics studies. Previous research has shown that the SSA technique is very effective in signal extraction and noise filtering in genetics data.

Theoretical developments presented here as two-dimensional SSA and SSA based on minimum variance have also enabled the researchers to achieve enhanced results and a better estimation of extracted signal. As a non-parametric method SSA has given us very promising results which are more reliable than those obtained by other methods. However, SSA has not revealed its full potential yet, areas like optimising the embedding dimension and change point detection are still open to pursue.

## Footnotes

1 The SVD algorithms used in this paper are either based on Hankel or Teoplitz matrix.

## References

[1] Allison D.B., Cui X., Page G.P., Sabripour M. (2006). Microarray data analysis: from disarray to consolidation and consensus. Nat Rev Genet.

[2] Goeman J.J., Bhlmann P. (2007). Analyzing gene expression data in terms of gene sets: methodological issues. Bioinformatics.

[3] VanGuilder H.D., Vrana K.E., Freeman W.M. (2008). Twenty-five years of quantitative PCR for gene expression analysis. Biotechniques.

[4] Bustin S.A. (2014). The reproducibility of biomedical research: sleepers awake!. BDQ.

[5] Huggett J., O’Grady J., Bustin S. (2014). How to make mathematics biology's next and better microscope. BDQ.

[6] Kruglyak L., Daly M.J., Reeve-Daly M.P., Lander E.S. (1996). Parametric and nonparametric linkage analysis: a unified multipoint approach. Am J Hum Genet.

[7] Grimm O., Coppey M., Wieschaus E. (2010). Modelling the bicoid gradient. Development.

[8] Golyandina N., Nekrutkin V., Zhigljavsky A.A. (2010). Analysis of time series structure: SSA and related techniques.

[9] Hassani H. (2007). Singular spectrum analysis: methodology and comparison. JDS.

[10] Sanei S., Ghodsi M., Hassani H. (2011). An adaptive singular spectrum analysis approach to murmur detection from heart sounds. IPEM.

[11] Xie H.B., Guo T., Sivakumar B., Liew A.W.C., Dokos S. (2014). Symplectic geometry spectrum analysis of nonlinear time series. Proc R Soc A.

[12] Du L.P., Wu S.H., Liew A.W.C., Smith D.K., Yan H. (2008). Spectral analysis of microarray gene expression time series data of *Plasmodium falciparum*. IJBRA.

[13] Tang V.T., Yan H. (2012). Noise reduction in microarray gene expression data based on spectral analysis. IJMLC.

[14] Broomhead D.S., King G.P. (1986). Extracting qualitative dynamics from experimental data. Physica D.

[15] Vautard R., Yiou P., Ghil M. (1992). Singular-spectrum analysis: a toolkit for short, noisy chaotic signal. Physica D.

[16] Ghil M., Taricco C., Castagnoli G.C., Provenzale A. (1997). Advanced spectral analysis methods. Past and present variability of the solar-terrestrial system: measurement, data analysis and theoretical model.

[17] Elsner J.B., Tsonis A.A. (1996). Singular spectral analysis. A new tool in time series analysis.

[18] Danilov D. (1997). Principal components in time series forecast. J Comp Graph Stat.

[19] Holloway D.M., Harrison L.G., Kosman D., Vanario Alonso C.E., Spirov A.V. (2006). Analysis of pattern precision shows that Drosophila segmentation develops substantial independence from gradients of maternal gene products. Dev Dyn.

[20] Spellman P.T., Sherlock G., Zhang M.Q., Iyer V.R., Anders K., Eisen M.B. (1998). Comprehensive identification of cell cycleregulated genes of the yeast *Saccharomyces cerevisiae* by microarray hybridization. Mol Biol Cell.

[21] Alexandrov T., Golyandina N., Spirov A. (2008). Singular spectrum analysis of gene expression profiles of early Drosophila embryo: exponential-in-distance patterns. Res Lett Signal Process.

[22] Gregor T., Wieschaus E.F., McGregor A.P., Bialek W., Tank D.W. (2007). Stability and nuclear dynamics of the bicoid morphogen gradient. Cell.

[23] Hilfinger A., Paulsson J. (2011). Separating intrinsic from extrinsic fluctuations in dynamic biological systems. PNAS.

[24] Klebanov L., Yakovlev A. (2007). How high is the level of technical noise in microarray data. Biol Direct.

[25] http://cran.r-project.org/web/packages/Rssa/Rssa.pdf.

[26] Surkova S., Kosman D., Koslov K., Manu E., Myasnikova A., Samsonova A. (2007). Characterization of the Drosophila segment determination morphome. Dev Biol.

[27] Lopes F.J.P., Vieira F.M.C., Holloway D.M., Bisch P.M., Spirov A.V. (2008). Spatial bistability generates hunchback expression sharpness in the Drosophila embryo. PLoS Comput Biol.

[28] Hassani H., Ghodsi Z. (2014). Pattern recognition of gene expression with singular spectrum analysis. Med Sci.

[29] Hassani H. (2010). Singular spectrum analysis based on the minimum variance estimator. Nonlinear Anal: Real World Appl.

[30] Liew A.W.C., Law N.F., Cao X.Q., Yan H. (2009). Statistical power of Fisher test for the detection of short periodic gene expression profiles. Pattern Recogn.

[31] Liew A.W.C., Xian J., Wu S., Smith D., Yan H. (2007). Spectral estimation in unevenly sampled space of periodically expressed microarray time series data. BMC Bioinform.

[32] Yeung L.K., Szeto L.K., Liew A.W.C., Yan H. (2004). Dominant spectral component analysis for transcriptional regulations using microarray time-series data. Bioinformatics.

[33] Liew A.W.C., Yan H. (2009). Reliable detection of short periodic gene expression time series profiles in DNA microarray data. International conference SMC.

[34] Gan X., Liew A.W.C., Ya H. (2006). Microarray missing data imputation based on a set theoretic framework and biological consideration. Nucleic Acids Res.

[35] Bozdech Z., Llinas M., Pulliam B.L., Wong E.D., Zhu J.C., DeRisi J.L. (2003). The transcriptome of the intraerythrocytic developmental cycle of *Plasmodium falciparum*. PLoS Biol.

[36] Tang T.Y., Yan H. (2010). Identifying periodicity of microarray gene expression profiles by autoregressive modeling and spectral estimation. Ninth international conference on machine learning and cybernetics.

[37] Tang T.Y., Liew A.W.C., Yan H. (2010). Periodicity analysis of DNA microarray gene expression time series profiles in mouse segmentation clock data. Stat Interface.

[38] Tang T.Y., Liew A.W.C., Yan H. (2010). Analysis of mouse periodic gene expression data based on singular value decomposition and autoregressive modeling.

[39] Tang T.Y., Yan H. (2012). Noise reduction in microarray gene expression data based on spectral analysis. Int J Mac Learn Cybern.

[40] Vikalo H., Hassibi B., Hassibi A. (2008). Modeling and estimation for real-time microarrays. IEEE J Sel Topics Signal Process.

[41] Rau A., Jaffrezic F., Foulley J.L., Doerge R.W. (2010). An empirical Bayesian method for estimating biological networks from temporal microarray data. Stat Appl Genet Mol Biol.

[42] Bremer M., Doerge R.W. (2009). The KM-algorithm identifies regulated genes in time series expression data. Adv Bioinform.

[43] Zhang J., Hassani H., Xie H., Wang S. (2013). Estimating multi-country prosperity index: a two-dimensional singular spectrum analysis approach. J Syst Sci Complex.

[44] Golyandina N.E., Usevich K.D. (2010). 2D-extension of singular spectrum analysis: algorithm and elements of theory. Matrix methods: theory, algorithms.

[45] Porcher A., Dostatni N. (2010). The bicoid morphogen system. Curr Biol.

[46] Spirov A.V., Holloway D.M. (2003). Making the body plan: precision in the genetic hierarchy of Drosophila embryo segmentation. In Silico Biol.

[47] Holloway D.M., Lopes F.J., da Fontoura Costa L., Travençolo B.A., Golyandina N., Usevich K. (2011). Gene expression noise in spatial patterning: hunchback promoter structure affects noise amplitude and distribution in Drosophila segmentation. PLoS Comput Biol.

[48] http://mesord.sourceforge.net.

[49] Holloway D.M., Spirov A.V. (2011). Gene expression noise in embryonic spatial patterning. 21st international conference on noise and fluctuations.

[50] Golyandinaa N.E., Hollowayb D.M., Lopesc F.J.P. (2012). Measuring gene expression noise in early Drosophila embryos: nucleus-to-nucleus variability. Proc Comput Sci.

